# Label-Free Single-Molecule Conalbumin Analysis

**DOI:** 10.3390/mi17010094

**Published:** 2026-01-11

**Authors:** Tianyu Zhao, Xi Ren, Reuven Gordon

**Affiliations:** Department of Electrical Engineering, University of Victoria, Victoria, BC V8W 2Y2, Canada

**Keywords:** label-free single molecule sensing, nanoaperture optical tweezers, conalbumin

## Abstract

Nanoaperture optical tweezers (NOTs) were used to analyze conalbumin in various forms. By analyzing the power spectrum of the NOT-transmitted laser signal, differences between iron and iron-free conalbumin were observed; the corner frequency extrapolated to zero-laser power was significantly larger in magnitude for conalbumin with iron, which was interpreted as coming from the enhanced electrostatic interactions close to the surface of the nanoaperture. Conalbumin in a diluted, but otherwise unprocessed, egg white sample showed the same behavior as purified iron-free conalbumin. Dynamic two-state transitions in the NOT signal were observed for iron-free conalbumin and conalbumin in egg white samples. We used this to determine the dominant state as a function of temperature, with one state showing a maximum occupancy around 30.4 °C. Deconvolution of the probability distribution function was used to find the energy landscape associated with this two-state transition. This work shows the potential of NOTs to see variations with metal ion binding, including conformational dynamics related to the binding at timescales not accessible to other methods.

## 1. Introduction

**Figure 1:**
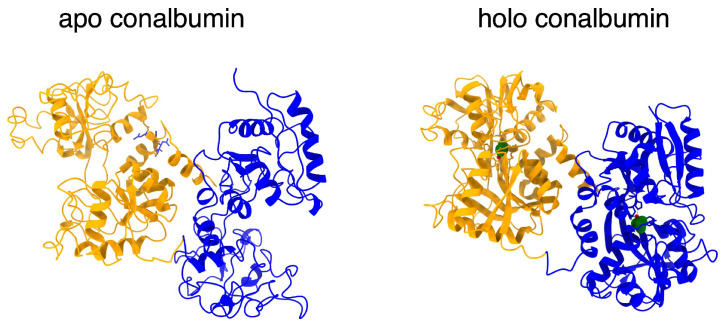
Structural models of conalbumin in its open apo (**left**) (PDB: 1AIV) and closed (**right**) holo forms (PDB: 10VT).

Conalbumin, also known as ovotransferrin, is a 76 kDa iron-transporting protein found in eggs. There have been many structural characterizations of transferrins [1], and two primary conformations have been identified, apo and holo, where holo is the iron-bound form and apo is the iron-free form, as shown in Figure 1. The structure of diferric holo conalbumin was resolved by X-ray crystallography [2], and this was later achieved for the apo ovotransferrin [3]. Ovotransferrin plays a role as an iron transporter, functions to prevent infection, and can serve as a biomarker for inflammation and other conditions [4].

Here we study conalbumin with nanoaperture optical tweezers (NOTs). NOTs have been used to trap dielectric objects in the single-digit nanometer range (including single proteins) in previous studies [5,6,7,8,9,10,11,12,13,14,15,16,17,18,19,20,21,22,23]. Previously, we studied conalbumin with NOTs, using thermal motion fluctuations to deduce the mass of different purified sample proteins (from the amplitude and autocorrelation time constant) [24]. In that work, it was found that conalbumin exhibited a bimodal distribution in the NOT laser transmission signal representing two distinct states, but further analysis of this behavior was not carried out and the dynamics of transitions between those states was not studied. Later, we trapped conalbumin as one of the proteins in unpurified (diluted) egg white samples, where it was shown that it could be distinguished from the other proteins in the sample, allowing for composition analysis [25].

More recently, we explored the dynamic conformational transitions of bovine serum albumin, seen in our first single-protein trapping studies [6], to deduce the conformational energy landscape of that protein [26,27]. We also extrapolated the NOT corner frequency to zero power to determine electrostatic surface interactions of proteins (and thereby probe their charge state) [28]; however, conalbumin was not studied in that work.

In this work, we apply these characterization approaches to conalbumin. Specifically, we look at the conformational transitions of conalbumin for samples that have iron and are iron-free, and those derived from diluted (but otherwise unprocessed) egg white. We note differences in the electrostatic interactions with iron state as found from the zero power corner frequency. We observe the dynamics of state transitions for the iron-free conalbumin. From the probability distribution function, the energy landscape is found by deconvolution and the Gibbs free energy difference between the two prominent states is extracted. Maximum stability is found for the one state at 30.4 °C, using a similar approach as applied to bovine serum albumin in the past [27].

## 2. Nanoaperture Optical Tweezer Method

### 2.1. Optical Trapping Setup

**Figure 2:**
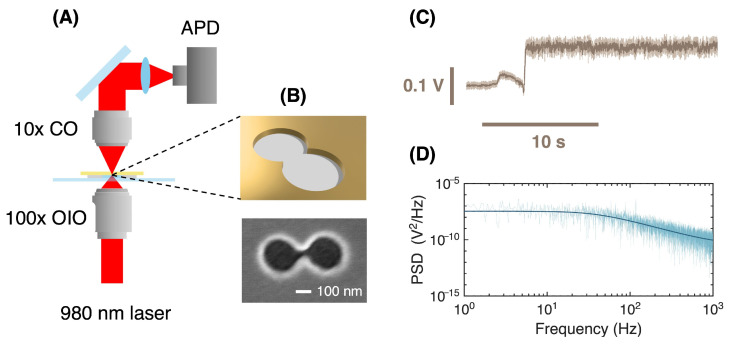
Experimental configuration and an example of the trapping event. (**A**) Schematic of NOT setup and (**B**) top—schematic of double nanohole structure, bottom—a scanning electron microscope image of the DNH. (**C**) An example of the NOT trapping of conalbumin with iron. (**D**). The power spectral density and Lorentzian fit of the trapping signal.

Figure 2A shows a schematic of the NOT setup. The 980 nm laser was collimated out of the fiber to form a beam size of 2 mm and passed through a linear polarizer and half-wave plate to control the orientation of polarization to maximize the detected power. The laser was then expanded with a beam expander and incident on a 100×, 1.25 numerical aperture oil immersion objective (Nikon E-plan, NJ, USA), filling the aperture of the objective. The light transmitted through nanoaperture was collected by a 10× microscope objective (Nikon MRP70100, NJ, USA) and focused onto an avalanche photodiode (Thorlabs APD120A, NJ, USA). A piezo stage was aligned with the nanoaperture for the maximum transmission of laser light. We have shown that the local temperature increases due to heating from a 980 nm laser by 0.58 K/mW [26]. In the same manner, the local temperature of the protein in this work was varied by changing the laser power from 6.8 mW to 11.3 mW. The temperature accuracy based on this conversion was better than 0.1 °C.

### 2.2. Colloidal Lithography of Double Nanoholes

Figure 2B shows a schematic of the double nanohole (DNH) aperture and a scanning electron microscope (SEM) image of the DNH. To prepare these samples, we followed the approach of past works [29]. Briefly, glass slides $\left(76\;\times \;25.4\;\times \;1\;{\mathrm{mm}}^{3}\right)$ were divided into thirds using a diamond scribe. The slides were then bathed in ethanol and sonicated for 10 min, followed by rinsing with acetone, deionized water, and ethanol, and drying with nitrogen between each step. A solution was made of 20 μL of 300 nm polystyrene beads and 1 mL of ethanol. Using 10 μL of the polystyrene–ethanol solution, a zig–zag pattern was deposited on the microscope slides and left overnight for drying. Once the ethanol evaporated, the slides were put into an oxygen plasma machine (Harrick PDC, NY, USA) for 110 s to reduce the size of the gap between the cusps of the DNH. To ensure the reproducibility of aperture size and shape, the slides were etched individually in the same position inside the machine, in order to achieve consistent plasma coverage. The slides were then coated with 7 nm titanium and 70 nm gold by a sputter deposition system (Mantis QUBE, UK). Polystyrene beads were removed by bathing in ethanol and sonicating for 8 min. A flowchart of the NOT sample preparation is given in past works [29,30]. This approach yielded approximately 4 DNHs in the microscope’s field of view. To investigate the influence of surface interactions, coating with a mPEG-thiol was applied to a portion of the samples. For these samples, a solution was made of 32 mg 8000-PEG-thiol (Sigma-Aldrich, ON, Canada P2139) and 20 mL 95% ethanol. The sample was immersed in the solution for 16 h and then stored at 4 °C to keep the surface coating stable [31].

### 2.3. Protein Solutions

The protein solutions were made by dissolving solid proteins or diluting liquid egg white in phosphate buffered saline (PBS). Pure conalbumin samples with iron (Sigma-Aldrich C7786) and without iron (Sigma-Aldrich C0755) were prepared at 1 mg/mL in PBS. The egg white trapping solution was made of 20 μL consumer egg white (Vanderpol’s) and 1.08 mL PBS solution. All trapping solutions were stored in 20 μL aliquots at −20 °C.

### 2.4. Data Acquisition and Analysis

Figure 2C shows a typical trapping event, noted by a jump in the transmitted laser intensity and an increase in the noise coming from thermal motion of the trapped protein. The trapping data was recorded by a data acquisition card (Omega USB-471A, QC, Canada) with a sampling rate of 100 kHz. All data analysis was conducted using custom MATLAB, R2022a code (available on github, link provided in Data Availability section). Figure 2D shows the power spectral density (PSD) of the signal after trapping following a detrend function to remove linear drift and the DC offset. The corner frequencies were found by calculating the power spectrum densities and fitting with a Lorentzian. By the Wiener–Khinchin theorem, the corner frequency is the inverse of the exponential decay time constant found via autocorrelation analysis, as applied in optical tweezer experiments in the past [24,32,33]. Here we use PSD analysis instead of autocorrelation to avoid artifacts from line noise and other sources that lead to ripple in the signal. The corner frequency occurs where the Lorentzian is reduced by 3 dB from its low frequency value. Corner frequencies in laser tweezer experiments are used routinely to characterize the stiffness of the trap, since the fluctuations come from the thermal motion of the trapped particle in the optical potential [34]. For each power, a 10 s data segment was used for the PSD calculation.

State recognition was achieved by selecting transitions based on the first derivative of the low-pass filtered signal. An abrupt change was considered statistically significant when the derivative value exceeded the overall standard deviation of the absolute derivative values by a factor of 3. Genuine steps were distinguished from transient noise by analyzing the local standard deviation within a 0.1 s window.

Energy landscapes were calculated by three steps. First, the probability density function (PDF) was found by applying a dithering technique (adding random noise with an amplitude of the least significant bit 0.002441 V) to eliminate digitalization artifacts. Second, the observed PDF was processed via Lucy–Richardson deconvolution (51 iterations) with a Gaussian point spread function (σ≈0.0075 V, corresponding to 6% of the mean value) to remove broadening from thermal motion and not attributed to conformational changes (i.e., translation and rotation in the optical trapping potential) [26]. σ was found by the standard deviation for a single state which was dominated by thermal motion (the laser noise was 20 times smaller). Finally, the deconvolved distribution was converted to free energy via $U\left(V\right)=-k_{B}T\ln\left[P\left(V\right)\right]$. This free energy is interpreted as the Gibbs free energy incorporating both enthalpy and entropy, as is typical when using the Boltzmann inversion [35]. The Gibbs free energy difference, ΔG, was then determined by the difference in energy values between the identified local minima of the landscape.

## 3. Results and Discussion

### 3.1. Extrapolated Corner Frequency

**Figure 3:**
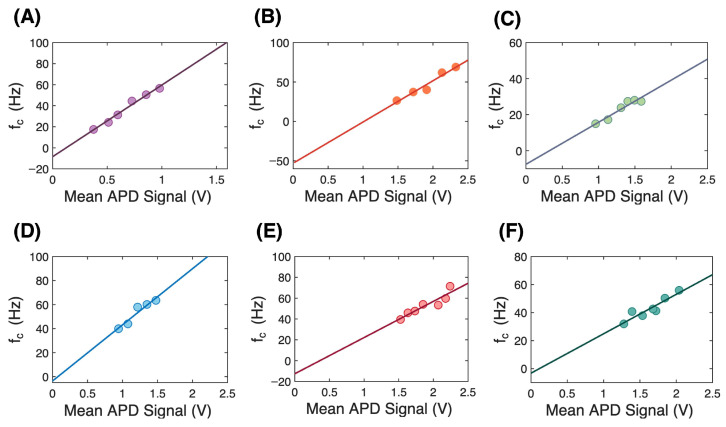
Conalbumin trapping corner frequency used to extrapolate a zero-power value. (**A**) Pure conalbumin without iron (−8.44 Hz), (**B**) pure conalbumin with iron (−48.61 Hz), (**C**) conalbumin in egg white (−7.57 Hz), (**D**) pure conalbumin without iron and with PEG-thiol-coated gold sample (−3.6 Hz), (**E**) pure conalbumin with iron and with PEG-thiol-coated gold sample (−12.64 Hz), and (**F**) conalbumin in egg white with PEG-thiol-coated gold sample (−3.24 Hz).

Figure 3 shows the corner frequency as a function of the APD voltage (proportional to the transmitted laser power through the DNH). The APD dark count was lower than the least significant bit in data acquisition and so it appeared as zero and did not have to be removed. We extrapolate a linear fit to zero power to find the residual corner frequency that comes from interactions with the DNH environment. The corner frequency arises from a confining potential. When it is zero, this marks the transition to a repulsive potential. A negative corner frequency is a measure of how much confining potential must be added to the repulsive potential to cancel it out. This was previously used by our group to determine electrostatic interactions [28]. Here we apply the approach to conalbumin, without and with iron, and to conalbumin in egg white. We consider both the cases of bare gold and gold coated with mPEG-thiol.

The iron-containing conalbumin sample had a larger zero-power corner frequency than the iron-free sample. The egg white sample nominally had an identical zero-power corner frequency to the iron-free sample. This result agrees with past work that found that egg white conalbumin is iron-free and can be converted to the iron-containing form by the addition of iron [36].

The magnitude of the zero-power corner frequencies was reduced by the mPEG-thiol coating; however, the iron-containing version still had a larger magnitude zero-power corner frequency. The surface coating increases the separation between the protein and the gold surface to around 10 nm, and thereby reduces the electrostatic interaction as reflected in the measurements [37].

The iron-containing protein has a lower isoelectric point of 5.78 (as compared with 6.73 for the iron-free version) and a larger negative charge at the PBS buffer pH of 7.4 [38]. Conformational changes also perturb the charge distribution that could also impact interactions with the surface [39]. The zero-power corner frequency removes the laser power dependence and so it does not have a contribution from the polarizability of the protein, which also changes with conformation [40]. The larger charge gives a larger electrostatic interaction and this was found in the observed magnitude of the zero-power corner frequency. Other than the addition of ion charge and the conformational change of the molecule, the exact mechanism of the shift in the zero-power corner frequency with iron binding requires further investigation.

### 3.2. Dynamic Transitions

**Figure 4:**
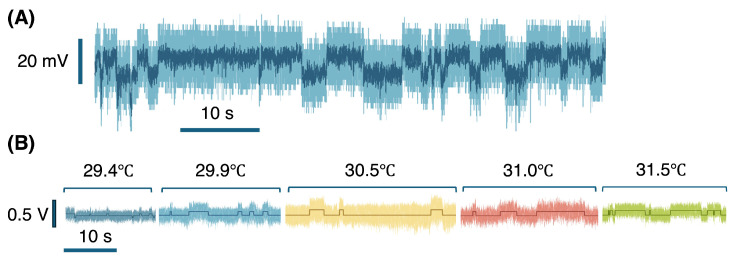
State transition of iron-free conalbumin. (**A**) Transmission through DNH at 29.9 °C. Light blue shows signal at 100 kHz sampling rate, and the dark blue signals are 10 Hz low-pass filtered signals. (**B**) State transition of iron-free conalbumin without mPEG-thiol surface coating at different temperatures.

Figure 4A shows the APD voltage after trapping for conalbumin without iron. The signal shows repeated stochastic transitions between two discrete states. We attribute these transitions to conformational changes in the iron-free form changing the light scattering of the protein, as was observed previously for bovine serum albumin [26,40]. We confirmed that the protein was not sticking to the surface by blocking and then unblocking the laser by seeing that the APD voltage return to the untrapped value, showing that the protein was released. Stepping behavior was observed consistently for the uncoated gold samples, but was also observed in some cases for mPEG-thiol-coated samples. We believe that the electrostatic interactions with the surface orient the protein and allow for the conformational changes to the observed, whereas when the protein is not close to the surface, this orientation dependence is diminished. It is well studied that conalbumin transitions between holo and apo forms with the release of iron. Our studies suggest that an analogous intermittent conformational transition is possible even in the absence of iron for the apo form, whereas the holo state is stably in a single conformation. The conformational transition leads to changes in optical scattering that are observed in our NOT experiment [40].

To further explore the dynamics of this state transition, we increased the laser power to change the local temperature, as shown in Figure 4B. State recognition was applied to this data to produce the steps shown in the figure.

**Figure 5:**
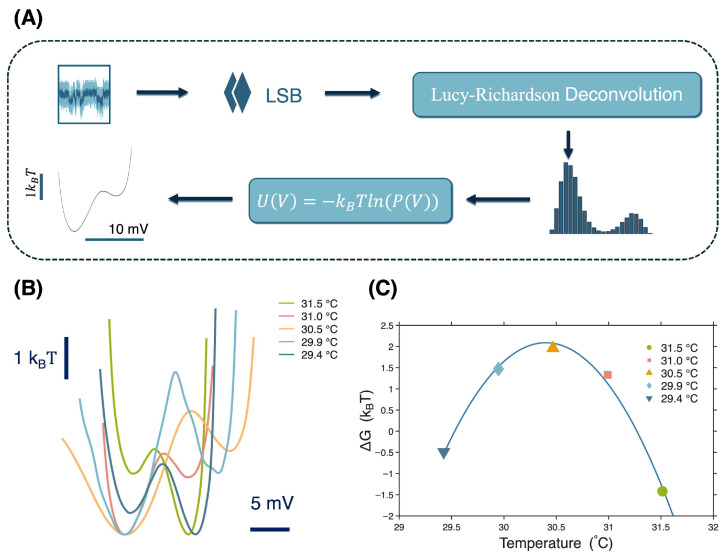
(**A**) Flow chart of free energy landscape generation. (**B**) Energy landscapes of the trapping signals at different temperatures (31.5 °C, 31.0 °C, 30.5 °C, 29.9 °C, and 29.4 °C). (**C**) Gibbs free energy difference between two states at different temperatures.

Figure 5A shows the procedure for generating the free energy landscape by dithering, deconvolution, and an inverse Boltzmann method. Figure 5B shows the energy landscapes with different temperatures. The higher voltage state (corresponding to a higher optical polarizability and therefore more light scattering and optical transmission [40]) was at the lower Gibbs free energy value for the lowest and highest temperatures. In the intermediate temperature range, the lower voltage state had the lower energy and was more probable. Since extended particles are more polarizable, we attribute the higher voltage state to the open apo form of the protein. It seems from this observation that the iron-free protein actually transitioned between an open and a more compact form, dynamically. It is not possible to observe these transitions in the static crystalline structure of the protein [1,2].

Figure 5C shows the evolution of the change in Gibbs free energy between the two states as a function of temperature. This shows a maximum stability for the lower voltage state at the temperature of 30.4 °C (using a second-order fit). We previously observed a similar maximum stability for the conformational states of bovine serum albumin at lower temperatures [27]. There have been studies related to temperature-dependent conformational sampling and how this impacts the function of the protein [41]. In the future, it would be interesting to explore how this maximum stability impacts the ability of conalbumin to sequester iron.

In this work, we observed the same transition behavior for egg white-derived conalbumin, but for conalbumin with iron, we consistently observed two discrete steps of increasing voltage, after which no further transitions were seen. The pure iron-free conalbumin displayed the same behavior as conalbumin in egg white. It is possible that the native biological environment does modify the behavior of this protein and further studies would be required to show such effects.

The egg white sample contains other proteins, and the abundance of conalbumin is 11% [42]. We conducted clustering of the egg white trapping data based on corner frequency and the amplitude of voltage fluctuations and found that 5 out of 55 trapping events were attributed to conalbumin, also showing the two-state transitions observed here and in past works [24,25]. Events with corner frequencies in the range of 36 Hz to 56 Hz (for 9 mW of power), were attributed to conalbumin, whereas the majority of the events had a lower corner frequency and were attributed to ovalbumin. The corner frequency is inversely related to the autocorrelation time constant used to separate out the different components of egg white in a previous work [25].

## 4. Conclusions

The zero-power extrapolated corner frequency was demonstrated as a method to distinguish between iron-containing and iron-free forms of conalbumin, and to determine that the egg white conalbumin is iron-free. Past works have also noted that conalbumin in egg white is mainly iron-free, which allows it to sequester iron and limit infection [36,39]. The iron-free form of conalbumin transitions between two states, and we applied a deconvolution approach to map out the energy landscape of these transitions as a function of temperature. This dynamic behavior provides new insight into structural fluctuations of the iron-free form not possible from static measurements. It appears that the protein does not exist purely in a single apo form, but rather transitions between an open and a closed state in the absence of iron. The lower-voltage (closed) state can actually be the more stable configuration for temperatures close to 30.4 °C.

Future work could extend these findings by adding microfluidics to the setup [31]. In that case, we would be able to dynamically change the pH to release iron, or introduce iron to the iron-free form to directly observe the binding of iron to the protein. Changes in pH and iron have been observed in previous studies using NOTs with ferritin, including microfluidics [22,43]. The kinetics of the interaction of conalbumin with iron have previously been investigated showing complicated behavior [44]. The time resolution of the NOT approach may make it possible to resolve the dynamic processes that occur at faster than 5 ms timescales not accessible to previous works [44]. In particular, Figure 2D shows that the frequency roll-off extends to kHz without hitting the noise floor, so millisecond dynamics are resolvable within our current setup. It is possible that improving the sensitivity and reducing the laser source noise will make it possible to observe changes at the microsecond or faster timescale relevant to conformational changes [40]. Optical torque is also possible to observe in optical tweezers and it may be interesting to study interactions with chiral biomolecules [45,46].

## Data Availability

Data and Matlab routines used in analysis can be found at http://github.com/nanoplasmonics/ovotransferrin (accessed 8 January 2026).

## Abbreviations

The following abbreviations are used in this manuscript:

NOT	Nanoaperture optical tweezer
DNH	Double nanohole
APD	Avalanche photodiode
PBS	Phosphate-buffered saline
PSD	Power spectral density
PDF	Probability density function
PEG	Polyethylene Glycol
ΔG	Gibbs free energy difference
$k_{B}$	Boltzmann constant
